# Early fish introduction and neonatal antibiotics affect the risk of asthma into school age

**DOI:** 10.1111/pai.12078

**Published:** 2013-04-11

**Authors:** Emma Goksör, Bernt Alm, Rolf Pettersson, Per Möllborg, Laslo Erdes, Nils Åberg, Göran Wennergren

**Affiliations:** Department of Paediatrics, Queen Silvia Children's Hospital, University of GothenburgGothenburg, Sweden

**Keywords:** antibiotics, asthma, atopic asthma, cohort study, fish

## Abstract

**Background:**

The early introduction of fish has been reported to reduce the risk of wheezing disorder in early childhood, while broad-spectrum antibiotics in the first week have been associated with an increased risk. However, it is uncertain whether the effects remain into school age. The aim was to explore these risk factors for doctor-diagnosed asthma at 8 years.

**Methods:**

Data were obtained from a prospective, longitudinal study of a cohort of children born in western Sweden. The parents answered questionnaires at 6 months and 1, 4.5 and 8 years of age. The response rate at 8 years was 80% of the questionnaires distributed (4051/5044), that is, 71% of the families entering the study (4051/5654).

**Results:**

At 8 years, 5.7% reported current doctor-diagnosed asthma. Of these, 65% had atopic asthma and 35% non-atopic asthma. In the multivariate analysis, atopic heredity, male gender and own allergic disease during infancy were risk factors for doctor-diagnosed asthma at 8 years. In addition, the introduction of fish before the age of 9 months independently reduced the risk (adjusted OR 0.6; 95% CI 0.4–0.96), while broad-spectrum antibiotics in the first week independently increased the risk of current asthma at school age (aOR 2.3; 1.2–4.2). Regarding types of asthma, the effects were significant in atopic asthma but not in non-atopic asthma.

**Conclusion:**

The early introduction of fish and neonatal antibiotic treatment influence the risk of asthma into school age. The significant effect on atopic asthma is of particular importance, as this phenotype is of major clinical significance.

The detection of early life factors affecting the risk of asthma can increase our understanding of pathophysiologic mechanisms. In addition, these events can become targets for intervention with a long-term impact.

Both high consumption and early introduction of fish have been suggested to reduce the risk of allergy and asthma development 1–8. A protective effect on the risk of recurrent wheeze at preschool age has been reported 7. However, wheezing during the first years of life is a heterogeneous disorder and the prognosis varies 9. By school age, the diagnosis of asthma is more reliable. At school age, especially atopic asthma is associated with an increased risk of adult asthma 10.

Antibiotic treatment disturbing the intestinal microbiota in early life might affect the maturation of the immune system and increase the risk of subsequent allergic disease 11. Treatment with antibiotics during the first year of life has been associated with an increased risk of wheeze, asthma and allergic disease in infancy and at preschool age 7, 11–14. However, prospective studies up to school age are rare and the possibility of confounding has to be considered 14–16.

The aim of this study was to explore the impact of early life events on the risk of asthma at 8 years, with special reference to the early introduction of fish and treatment with antibiotics neonatally. To avoid reverse causation, only broad-spectrum antibiotics given during the first week of life were considered. In addition, the risk factors for atopic and non-atopic asthma were explored.

## Methods

### Participants

Data were obtained from a prospective, longitudinal cohort study of children born in the region of western Sweden in 2003. The random sample comprised 8176 families (50% of the birth cohort), with 5654 families entering the study.

### Procedures

After written informed consent, the parents answered questionnaires at 6 months and 1, 4.5 and 8 years of age. The questionnaires were based on the Swedish version of ISAAC and the Swedish BAMSE study. Details regarding the questionnaires and earlier response rates have been published previously 3, 6, 7, 12. In addition, supplementation with data from the Swedish Medical Birth Register (MBR) was performed. At 8 years, questionnaires were distributed to the families entering the study (n = 5654), except for those that had declared that they no longer wished to participate (n = 610). The response rate at 8 years was 80.3%, that is, 4051 of the 5044 questionnaires distributed. This equals 49.5% of the families that were initially contacted, 71.6% of the families entering the study and 83.5% of the responders at 4.5 years of age. Thus, the study population at 8 years consisted of 4051 responders, and 3487 of these had responded to all the previous questionnaires and the MBR.

Information regarding pregnancy and post-natal factors was collected at 6 months of age. Supplementary information regarding pregnancy and delivery was obtained from the MBR and gave information on caesarean section, gestational age, small for gestational age, large for gestational age, gender and Apgar score.

Information on admission to a neonatal ward during the first week of life and treatment with broad-spectrum antibiotics during this period was obtained from the 6-month questionnaire. Information regarding the duration of breastfeeding and the introduction of different foods including fish was collected at 12 months. Specific information regarding these questions has been published previously 7.

At 8 years, questions were asked regarding current health and disease, family, environment and feeding habits.

### Definitions

The diagnosis of current asthma at 8 years of age was based on the answers to the following questions:

Q1: ‘Has your child been diagnosed with asthma by a physician?’
Q2: ‘Has your child received medication for asthma during the last 12 months?’
Q3: ‘Has your child had problems with/symptoms of wheezing [‘pipande eller väsande’ breathing] during the last 12 months?’

Current asthma at 8 years was defined as a positive answer to Q1 (doctor diagnosed) and either or both Q2 (current asthma medication) and Q3 (current wheezing). Subjects with current asthma therefore had a doctor's diagnosis of asthma and either current treatment or symptoms.

Atopic asthma at 8 years was defined as current asthma, as stated above, and reported allergic sensitization and/or current doctor-diagnosed rhinoconjunctivitis, food allergy or eczema. A current doctor diagnosis was defined as reported diagnosis and either treatment and/or symptoms during the last 12 months. The questions regarding allergic diagnoses and symptoms are summarized in Table S1.

Non-atopic asthma at 8 years was defined as current asthma, as stated above, and not having allergic sensitization or current doctor-diagnosed rhinoconjunctivitis, food allergy or eczema.

### Statistical analyses

In the statistical analysis, contingency tables with the χ^2^ test and binary logistic regression were used. Odds ratios (OR) were estimated with 95% confidence intervals (CI). Crude ORs are indicated as ‘OR’ and adjusted ORs as ‘aOR’.

Factors considered in the univariate analyses are summarized in Table S2. Factors that were significant with a p-value of <0.1 in the univariate analysis (Table 1) were analysed in the multivariate model (Table 2). A cut-off of 0.1 was chosen so that potential confounders would be included. The multivariate model also controlled for maternal smoking during pregnancy and any breastfeeding for 4 months or more, as these factors have previously been reported to affect the risk of childhood asthma. In addition, parental level of education was included as a marker of socio-economic status. Adjustments were made for all factors simultaneously in one multivariate model.

**Table 1:** Risk factors with a significance level of p < 0.1 in the univariate analysis for current doctor-diagnosed asthma at 8 years of age (n = 4051)

Risk factor	Asthma n (%) 231 (5.7)	No asthma n (%) 3820 (94.3)	OR	95% CI
Atopic heredity (mother or father with asthma, eczema or rhinoconjunctivitis)	179 (77.5)	2263 (59.9)	2.3	1.7–3.2
Male gender	146 (63.5)	1970 (51.9)	1.6	1.2–2.1
Maternal medication during pregnancy	90 (39.1)	1037 (27.6)	1.7	1.3–2.2
Gestational age < 37 weeks	19 (8.4)	184 (4.9)	1.8	1.1–2.9
Caesarean section	43 (19.1)	526 (14.0)	1.4	1.02–2.0
Small for gestational age	8 (3.7)	57 (1.6)	2.4	1.1–5.1
Admission to a neonatal ward	36 (15.6)	403 (10.7)	1.5	1.1–2.2
Treatment with antibiotics during the first week	19 (8.2)	167 (4.4)	1.9	1.2–3.2
Doctor-diagnosed food allergy during the first year	36 (17.6)	142 (4.0)	5.1	3.4–7.6
Eczema during the first year	82 (39.4)	700 (19.8)	2.6	2.0–3.5
Introduction of fish before 9 months of age	136 (75.6)	2772 (84.5)	0.6	0.4–0.8
Fish once a month or more at 1 year of age	168 (82.0)	3286 (92.7)	0.4	0.2–0.5
Fermented food once a month or more at 1 year of age	166 (81.8)	3207 (90.9)	0.5	0.3–0.7
Choice of spread at 1 year of age				
Butter	122 (60.1)	2482 (70.7)	1	ref.
Margarine	70 (34.5)	846 (24.1)	1.7	1.2–2.3
No spread	11 (5.4)	181 (5.2)	1.2	0.7–2.3
Daily outdoor activity at 12 months				
<1 h	9 (4.3)	261 (7.4)	1	ref.
1–3 h	173 (83.2)	2718 (76.8)	1.8	0.9–3.7
>3 h	26 (12.5)	558 (15.8)	1.4	0.6–2.9
Father employment at 6 months	229 (99.1)	3624 (96.6)	4.1	1.002–16.6
Rural living at 6 months	40 (17.5)	834 (22.4)	0.7	0.5–1.04

**Table 2:** Multivariate analysis for current doctor-diagnosed asthma (n = 231), atopic (n = 150) and non-atopic asthma (n = 81) at 8 years of age

Risk factor	Current asthma aOR (95% CI)	Atopic asthma aOR (95% CI)	Non-atopic asthma aOR (95% CI)
Atopic heredity (mother or father with asthma, eczema or rhinoconjunctivitis)	**1.8 (1.2–2.7)**	1.5 (0.9–2.5)	**2.4 (1.2–5.0)**
Male gender	**1.7 (1.2–2.5)**	**1.7 (1.1–2.6)**	1.8 (1.0–3.4)*
Maternal medication during pregnancy	1.2 (0.9–1.8)	1.2 (0.8–1.9)	1.5 (0.8–2.7)
Gestational age < 37 weeks	1.4 (0.7–2.8)	1.0 (0.4–2.4)	**2.7 (1.1–6.7)**
Caesarean section	1.3 (0.8–2.1)	1.2 (0.7–2.2)	1.5 (0.7–3.1)
Small for gestational age	2.2 (0.9–5.7)	**3.2 (1.2–9.2)**	1.0 (0.1–8.3)
Treatment with antibiotics during the first week	**2.3 (1.2–4.2)**	**3.0 (1.5–6.0)**	1.3 (0.4–4.5)
Doctor-diagnosed food allergy during the first year	**4.2 (2.5–7.1)**	**5.9 (3.3–10.4)**	0.6 (0.1–4.7)
Eczema during the first year	**1.7 (1.1–2.5)**	**2.6 (1.6–4.0)**	0.6 (0.3–1.4)
Introduction of fish before 9 months of age	**0.6 (0.4–0.96)**	**0.5 (0.3–0.8)**	0.9 (0.4–2.1)
Fish once a month or more at 1 year of age	0.6 (0.3–1.2)	0.5 (0.3–1.0)	2.3 (0.3–17.4)
Daily outdoor activity at 12 months			
<1 h	1 ref.	1 ref	1 ref.
1–3 h	1.6 (0.7–3.5)	2.7 (0.8–8.8)	0.9 (0.7–3.5)
>3 h	0.9 (0.3–2.3)	1.3 (0.3–5.0)	0.6 (0.2–2.5)
Father employment at 6 months	2.0 (0.5–8.6)	1.3 (0.3–5.6)	†
Rural living at 6 months	1.0 (0.6–1.5)	1.0 (0.6–1.8)	0.9 (0.4–1.8)
Breastfeeding for 4 months or more	0.8 (0.5–1.2)	0.8 (0.5–1.4)	0.8 (0.4–1.6)
Smoking during pregnancy	1.2 (0.6–2.2)	1.4 (0.7–2.9)	0.6 (0.2–2.6)
Parental educational level (>12 years) at 6 months	1.0 (0.7–1.4)	0.8 (0.5–1.2)	1.7 (0.9–3.4)

Adjustments were made for all factors simultaneously. Independently significant aORs, p < 0.05, are in bold.

* 1.8 (1.0001–3.4).

† No OR is given due to too few index subjects.

The protective effect of the frequent consumption of fermented foods and the increased risk associated with margarine or ‘no spread’ at 12 months of age was closely associated with own food allergy in infancy (i.e., infants with cow's milk allergy avoiding yogurts and butter) and were therefore excluded from the multivariate model. Admission to a neonatal ward was closely related to receiving neonatal antibiotics and was also excluded.

Current doctor-diagnosed asthma at 8 years of age was used as the primary outcome variable. In addition, the multivariate analyses were performed for atopic and non-atopic asthma, respectively.

To identify not only large and obvious differences, but also minor but plausible differences, no correction for multiple testing was performed.

The IBM SPSS Statistics version 20.0 (IBM Corp., Armonk, NY, USA) was used for statistical calculations.

### Ethical approval

The study was approved by the ethics committee at the University of Gothenburg.

## Results

### Representativeness of the study sample

As reported earlier, the material is largely representative of the population 12. We analysed the differences between children who did not participate in this follow-up at 8 years but for whom we had data from the follow-up at 6 and 12 months. Among the non-responders, the frequency of parents with a low educational level, maternal smoking during pregnancy and preterm birth was higher. There also was a slightly lower prevalence of atopic heredity and breastfeeding. In addition, there was a somewhat higher frequency of recurrent wheeze in infancy among the non-responders (Table S3).

### Population characteristics

At 8 years of age, 5.7% (n = 231) reported current doctor-diagnosed asthma. Of these, 65% (n = 150) had atopic asthma and 35% (n = 81) non-atopic asthma. Treatment with asthma medication was reported in 9% (n = 309) of the total cohort, and of these, 65% reported treatment with inhaled corticosteroids and 11% reported treatment with a leukotriene antagonist. Wheeze during the last year was reported by 9.2% (n = 369).

### Univariate and multivariate analyses

Univariate risk factors with a p-value of <0.1 for current doctor-diagnosed asthma are shown in Table 1. The results of the multivariate analyses of risk factors for current doctor-diagnosed asthma, atopic asthma and non-atopic asthma are shown in Table 2.

### Early fish introduction

Introduction of fish before 9 months of age reduced the risk of doctor-diagnosed asthma at 8 years. The protective effect was seen in the children with atopic asthma but not in the children with non-atopic asthma (Table 2).

To analyse whether the effect of early fish introduction could be due to early risk reduction, the association with asthma with onset before and after 4.5 years was analysed. The association was significant for both groups (OR 0.5, 95% CI 0.3–0.8 and OR 0.6, 0.3–0.95, respectively).

Fish was introduced 1–2 weeks later in children with atopic heredity, eczema or doctor-diagnosed food allergy during infancy. However, we found no significant interaction between early fish introduction and parental atopic heredity (p = 0.61), eczema during infancy (p = 0.61) or food allergy in infancy (p = 0.52). Furthermore, we controlled for all these factors in the multivariate analyses.

### Neonatal antibiotics

Broad-spectrum antibiotics during the first week of life were an independent risk factor for atopic asthma but not for non-atopic asthma (Table 2).

The increased risk of asthma at 8 years of neonatal antibiotics was seen for those with an onset before but not after preschool age (OR 3.0, 95% CI 1.5–6.0 and OR 1.05, 0.4–2.7, respectively).

There was no significant interaction between atopic heredity or parental asthma and neonatal antibiotic treatment (p = 0.53 and p = 0.67, respectively). When controlling for parental asthma separately, the effect of neonatal antibiotic treatment still was independently significant. Furthermore, no significant interaction was seen between neonatal antibiotics and early fish introduction (p = 0.20).

### Atopic and non-atopic asthma

As seen in Table 2, the pattern of risk factors differed between children with atopic asthma compared to children with non-atopic asthma. Allergic sensitization in terms of a positive allergy test was reported in 84% of the subjects with atopic asthma.

## Discussion

In this prospective cohort study, we report a reduced risk of doctor-diagnosed asthma even at 8 years, following the early introduction of fish. Furthermore, treatment with antibiotics during the first week of life increased the risk. The associations were significant for atopic asthma but not for non-atopic asthma. We did no longer see any protective effect from breastfeeding for 4 months or more 12.

It has been suggested that the protective effect of fish can be attributed to the high content of n-3 polyunsaturated fatty acids (PUFA) in fish 8. There are some reports of a reduced risk of allergy and eczema in offspring following maternal high n-3 PUFA intake or supplementation with n-3 PUFAs during pregnancy 17, 18. However, this kind of supplementation in children with heredity for asthma did not prevent subsequent asthma development 19. There might therefore be other constituents of fish that account for the preventive effect. For example, fish contains vitamin D, which has also been proposed to reduce the risk of allergic disease 20.

The beneficial effect of fish on asthma and allergy development has been suggested to be explained by reverse causation. In this study, however, the protective effect of the early introduction of fish was independent of, and did not show any significant interactions with, own allergic disease during infancy or parental allergy and asthma. The association in our study can therefore hardly be explained by the later introduction of fish in children with early allergic manifestations or by allergic parents delaying introduction. This is further supported by the findings in a similar setting that the association between early fish consumption and preschool wheeze and eczema remained after excluding children with eczema during the first year of life 2. We found the effect of early fish introduction both on asthma with an onset before and after preschool age. Thus, the effect did not seem to be due to early risk reduction only. However, we do not know whether this is a long-lasting effect of early fish introduction or explained by early fish introduction being a proxy for continued high consumption of fish.

Finally, it has been suggested that the association is confounded by socio-economic and lifestyle factors 21. The multivariate model was therefore adjusted for such factors (Table 1).

Antibiotic treatment during the first year of life has been reported to increase the risk of subsequent wheeze and asthma 7, 11–14. However, there are not many prospective follow-ups into school age. In a recent systematic review, only three studies reported on asthma at school age and the authors called for more prospective studies 14.

There are findings that support a causal relationship between antibiotic treatment and subsequent asthma. Antibiotic treatment has been reported to change the intestinal microflora, disturbing the maturation of the immune system and affecting the development of immunologic tolerance 11. The immune response of the newborn is characterized by a Th2 response, and it has been suggested that the maturation towards a Th1 response is stimulated by microbial exposure in early life 22. It can be speculated that disturbances during this vulnerable period might be of greater importance than later disturbances 23. In line with these suggested mechanisms, we find an effect on atopic asthma but not on non-atopic asthma at school age.

The absence of effect of antibiotics on incident cases after 4.5 years indicates that the effect of early antibiotics occurred at early years but is still present.

A causal relationship between early antibiotics and subsequent wheeze and asthma has been questioned 14, 15. Children at risk of developing asthma and allergies have been reported to have a delayed immune maturation already at birth, as well as different cytokine profiles, increasing the risk of infections 24–26. As a result, the antibiotic treatment might be a marker of increased risk of infections and a disposition to develop asthma. To minimize confounding by post-natal vulnerability, we adjusted for parental allergy and asthma, as well as for preterm birth, caesarean section and being small for gestational age. To avoid reverse causation by treatment of early wheezing episodes with antibiotics, we only considered antibiotic treatment during the first week of life.

The different risk factor profiles for atopic and non-atopic asthma are largely in line with previous findings 27, 28. Atopic heredity increased the risk of current asthma and non-atopic asthma. However, for atopic asthma, it did not reach significance. The finding of an increased risk of atopic asthma among children born small for gestational age was somewhat unexpected. An association with reduced lung function and asthma has been suggested earlier, but a relation to allergy has been less obvious 29. However, a recent paper has reported results in line with ours 30. Atopic asthma was reported among 65% of the children with doctor-diagnosed asthma at school age. Children with atopic asthma tend to have more severe and persistent asthma 10. Factors associated with atopic asthma suggest an effect mediated via the immune system.

### Weaknesses and strengths

Questionnaire-based studies are accompanied by limitations relating to the validity and interpretation of the answers. To avoid this, we have used questions based on well-known, validated questionnaires. As our cohort was not clinically tested, we have defined subjects as having asthma if they reported a doctor's diagnosis of asthma and, in addition, had asthma medication and/or asthma symptoms. Likewise, the definitions of current rhinoconjunctivitis, eczema and food allergy required a doctor's diagnosis, in combination with current medication and/or symptoms. In addition, in absence of blood samples, the classification of atopic and non-atopic asthma was based on reported allergic sensitization or doctor-diagnosed allergic disease. Among the subjects with atopic asthma, 84% reported allergic sensitization. As is often seen in questionnaire studies, responders were somewhat more health conscious and educated compared with non-responders.

The strengths of this prospective follow-up include the large birth cohort size, access to perinatal data and the good response rate at school age.

## Conclusions

The early introduction of fish reduced the risk, while treatment with broad-spectrum antibiotics during the first week of life increased the risk of doctor-diagnosed asthma at 8 years of age. The associations were significant for children with atopic asthma but not for non-atopic asthma. The effect on atopic asthma is of major clinical importance and is suggestive of an effect mediated via the immune system.

## Supporting Information

**Table S1.** Questions on current allergic symptoms and diagnoses at age 8 years.

**Table S2.** Variables considered in the univariate analyses as potential risk factors for current doctor-diagnosed asthma at age 8 years.

**Table S3.** Responders at 8 years (n = 4051) vs. non-responders at age 8 years, that is, children who answered the questionnaire at 6 and/or 12 months of age but not at 8 years (n = 1602).
