# Supplementary material for: Early fish introduction and neonatal antibiotics affect the risk of asthma into school age
Source: Pediatr Allergy Immunol. 2013 Apr 11;24(4):339–44. doi: 10.1111/pai.12078 (PMC3712479; doi:10.1111/pai.12078)
Supplement: Supplementary file 1 [file pai0024-0339-SD1.docx]

*For the electronic repository*

**Table S1.** Questions on current allergic symptoms and diagnoses at age 8 years.

| Asked at age: | Consideration: | Question: |
| --- | --- | --- |
| 8 years | Allergic sensitisation | (i)“Has your child been tested with an allergy test (skin prick test or blood test)?”  Yes/no |
|  |  | (ii) If yes:  “What did the test show an allergic reaction to? (more than one answer is possible):  Airborne: dog, cat, horse, rabbit, dust mites, timothy, birch, mugwort, mould  Foods: egg, milk, fish, wheat, peanut, hazelnut, almond, other nuts, soy, pea” |
| 8 years | Current doctor-diagnosed rhinoconjunctivitis | “Has your child been diagnosed with hay fever or allergic sniffles (rhinitis) by a physician?”  Yes/no |
|  |  | “Has your child had symptoms of hay fever or allergic sniffles (sneezing, runny nose, nasal congestion, or red and itching eyes) during the last 12 months?”  Yes/no |
|  |  | “Has your child used medical treatment for hay fever in the last 12 months (tablets, nasal spray or eye drops) for these symptoms?”  Yes/no |
| 8 years | Current doctor-diagnosed eczema | “Has your child been diagnosed with eczema by a physician?”  Yes/no |
|  |  | “Has your child had symptoms of eczema during the last 12 months?”  Yes/no |
|  |  | “Has your child been treated with cortisone ointment for eczema during the last 12 months?”  Yes/no |
| 8 years | Current doctor-diagnosed food allergy | “Has your child been diagnosed with a food allergy by a physician?”  Yes/no |
|  |  | “Does your child have a doctor-diagnosed food allergy now?“  Yes/no |

*For the electronic repository*

**Table S2**: Variables considered in the univariate analyses as potential risk factors for current doctor-diagnosed asthma at age 8 years

|  |
| --- |
| Atopic heredity (mother or father with asthma, eczema or rhinoconjunctivitis) |
| Male gender |
| Maternal medication during pregnancy |
| Gestational age < 37 weeks |
| Caesarean section |
| Admission to a neonatal ward |
| Treatment with antibiotics during the first week |
| Doctor-diagnosed food allergy during the first year |
| Eczema during the first year |
| Introduction of fish before 9 months of age |
| Fish once a month or more at one year of age |
| Fermented food once a month or more at one year of age |
| Choice of spread at one year of age (butter, margarine, no spread) |
| Age at introduction of egg |
| Age at introduction of gluten |
| Age at introduction of purée |
| Symptoms of cow milk allergy during first year of life |
| Dog in the home |
| Cat in the home |
| Bird in the home |
| Rodent in the home |
| Alcohol use during pregnancy |
| Alcohol use in infancy (6 months) |
| Small for gestational age |
| Large for gestational age |
| Smoking during pregnancy |
| Smoking/moist snuff/nicotine supplementation during pregnancy |
| Parental smoking in infancy (6 months) |
| Maternal smoking in infancy (6 months)  Paternal smoking in infancy (6 months)  Daily outdoor activity at 6 months (less than one hour, one to three hours, more than three hours) |
| Daily outdoor activity at 12 months (less than one hour, one to three hours, more than three hours) |
| Choice of fat used in cooking at one year of age (butter, margarine, olive oil, other oils) |
| Choice of fish at one year of age (white fish, flatfish, herring/mackerel, salmon/game fish) |
| Choice of fatty fish at one year of age  Parental vegetarian food at one year of age |
| AD-vitamin supplementation at one year of age |
| Breast-feeding for 4 months or more |
| Asphyxia (Apgar < 7 at 5 min) |
| Parental educational level (> 12 years) at 6 months |
| Maternal employment at 6 months |
| Paternal employment at 6 months  Maternal age  Rural living at 6 months  Two or more siblings  Three or more siblings  Day care at 12 months |

*For the electronic repository*

## **Table S3.** Responders at 8 years (n=4,051) vs. non-responders at age 8 years i.e. children who answered the questionnaire at 6 and/or 12 months of age but not at 8 years (n=1,602).

| **Risk factor** | **Responders**  **n (%)**  4,051 (71.7) | | **Non-responders**  **n (%)**  1,602 (28.3) | | **p** |
| --- | --- | --- | --- | --- | --- |
| Atopic heredity (mother or father with asthma, eczema or rhinoconjunctivitis) |  | 2,452 (61) |  | 869 (55) | <0.001 |
| Parental educational level (>12 years) at 6 months |  | 2,494 (62) |  | 695 (44) | <0.001 |
| Male gender |  | 2,127 (53) |  | 733 (51) | 0.26 |
| Maternal smoking during pregnancy |  | 308 (8) |  | 228 (15) | <0.001 |
| Maternal medication during pregnancy |  | 1,131 (28) |  | 449 (29) | 0.69 |
| Gestational age < 37 weeks |  | 204 (5) |  | 90 (7) | 0.014 |
| Caesarean section |  | 571 (14) |  | 184 (14) | 0.96 |
| Treatment with antibiotics neonatally |  | 187 (5) |  | 66 (4) | 0.52 |
| Breast-feeding 4 months or more |  | 2,696 (80) |  | 749 (73) | <0.001 |
| Wheeze during the first year |  | 735 (20) |  | 260 (22) | 0.07 |
| Recurrent wheeze during the first year |  | 187 (5) |  | 78 (7) | 0.032 |
| Doctor-diagnosed food allergy during the first year |  | 178 (5) |  | 63 (5) | 0.44 |
| Eczema during the first year |  | 784 (21) |  | 249 (21) | 0.90 |
